# Experimental phasing for structure determination using membrane-protein crystals grown by the lipid cubic phase method

**DOI:** 10.1107/S1399004714010360

**Published:** 2015-01-01

**Authors:** Dianfan Li, Valerie E. Pye, Martin Caffrey

**Affiliations:** a Membrane Structural and Functional Group, School of Medicine and School of Biochemistry and Immunology, Trinity College Dublin, Dublin, Ireland

**Keywords:** co-crystallization, cysteine mutagenesis, heavy atoms, *in meso*, LCP, lipid mesophase, phase determination, pre-labelling, selenomethionine, soaking

## Abstract

Despite the marked increase in the number of membrane-protein structures solved using crystals grown by the lipid cubic phase or *in meso* method, only ten have been determined by SAD/MAD. This is likely to be a consequence of the technical difficulties associated with handling proteins and crystals in the sticky and viscous hosting mesophase that is usually incubated in glass sandwich plates for the purposes of crystallization. Here, a four-year campaign aimed at phasing the *in meso* structure of the integral membrane diacylglycerol kinase (DgkA) from *Escherichia coli* is reported. Heavy-atom labelling of this small hydrophobic enzyme was attempted by pre-labelling, co-crystallization, soaking, site-specific mercury binding to genetically engineered single-cysteine mutants and selenomethionine incorporation. Strategies and techniques for special handling are reported, as well as the typical results and the lessons learned for each of these approaches. In addition, an assay to assess the accessibility of cysteine residues in membrane proteins for mercury labelling is introduced. The various techniques and strategies described will provide a valuable reference for future experimental phasing of membrane proteins where crystals are grown by the lipid cubic phase method.

## Abstract 2

Very little information is available in the literature concerning the experimental heavy-atom phasing of membrane-protein structures where the crystals have been grown using the lipid cubic phase (*in meso*) method. In this paper, pre-labelling, co-crystallization, soaking, site-specific mercury binding to genetically engineered single-cysteine mutants and selenomethionine labelling as applied to an integral membrane kinase crystallized *in meso* are described. An assay to assess cysteine accessibility for mercury labelling of membrane proteins is introduced.

## 1. Introduction   

The 2012 Nobel Prize in Chemistry was awarded to Robert J. Lefkowitz and Brian K. Kobilka for studies on G protein-coupled receptors (GPCRs; Benovic, 2012 ▶). The crystal structure of the active β_2_-adrenergic receptor–G protein complex (Rasmussen *et al.*, 2011 ▶) was lauded by the Royal Swedish Academy of Sciences as a ‘molecular masterpiece’ and it figured as a prominent feature of the award-winning work. This structure was obtained using crystals grown by the lipid cubic phase (LCP) or *in meso* method. While interest in the method had been growing owing to its success in generating crystals and structures for a string of high-profile GPCRs and other important membrane proteins (Caffrey *et al.*, 2012 ▶), it was the Nobel Prize that really drew the attention of the community to the method.

The original observation that crystals of a membrane protein form in the cubic mesophase was reported almost two decades ago (Landau & Rosenbusch, 1996 ▶). Since that time, over 200 structures attributed to the *in meso* method have been deposited in the Protein Data Bank (PDB; http://www.rcsb.org). 97% of these were solved by molecular replacement. The first experimentally phased structure obtained from *in meso*-grown crystals was not reported until 2012. To date, only ten *in meso* structures have been phased experimentally (Fairman *et al.*, 2012 ▶; Kato *et al.*, 2012 ▶; Liao *et al.*, 2012 ▶; Doki *et al.*, 2013 ▶; Li, Lyons *et al.*, 2013 ▶; Wang *et al.*, 2013 ▶; Nogly *et al.*, 2014 ▶; Suzuki *et al.*, 2014 ▶; Xu *et al.*, 2014 ▶; Li *et al.*, 2014 ▶).

The *in meso* method makes use of a bicontinuous lipid mesophase, the cubic phase, in which the target protein is initially reconstituted. Crystals grow from within the cubic phase or a swollen variant thereof, the sponge phase (Caffrey, 2008 ▶). The mesophase itself is sticky and viscous, and handling it requires some manual dexterity and a few speciality tools. While there are a number of ways to go about experimental phasing, several involve treating the protein, pre- or post-crystallization, with hazardous heavy atoms. Doing this with the protein or crystal in a viscous mesophase and subsequent harvesting can be challenging, especially when glass sandwich crystallization plates (Caffrey & Cherezov, 2009 ▶) are used.

Given the success that the method has had with scientifically and medically important membrane proteins, more and more groups will wish to adopt it. Since most novel and thus high-profile membrane-protein targets are likely to have structures that cannot be phased by molecular replacement, experimental phasing will be required. Unfortunately, details of how to go about this in practice are sorely lacking. We needed to resort to experimental phasing to solve the structure of diacylglycerol kinase (DgkA; Li, Lyons *et al.*, 2013 ▶), an enzyme involved in phospholipid synthesis in the inner membrane of *Escherichia coli* (Van Horn & Sanders, 2012 ▶). The phasing aspect of the project took about three years of focused effort to complete. Here, we describe the many and varied approaches taken that finally led to a phased structure. These included heavy-atom pre-labelling, co-crystallization and crystal soaking, along with selenomethionine (SeMet) derivatization. Site-specific labelling of cysteine residues with mercury in single-cysteine mutants was also tested. How we went about the project, the methods that worked and those that did not and the lessons that were learned are detailed here. The information should prove useful to those embarking on a campaign to phase membrane-protein structures using crystals grown by the *in meso* method.

## 2. Materials and methods   

### 2.1. Materials   

The lipids used in this study included monoolein (9.9 MAG; Nu-Chek) and 7.8 MAG, which was synthesized and purified in-house following established procedures (Caffrey *et al.*, 2009 ▶; Yang *et al.*, 2012 ▶). Heavy-atom kits (Hampton Research) and Ta_6_Br_12_ (Jena Biosciences) were used for derivatization work. Details regarding the glass cutter and harvesting tools have been reported in an online open-access video article (Li, Boland, Aragao *et al.*, 2012 ▶). 5,5′-Dithiobis-2-nitrobenzoic acid (DTNB) was from Sigma. All other reagents for crystallization were sourced from Hampton Research.

### 2.2. Methods   

#### 2.2.1. Molecular cloning, protein purification and *in meso* crystallization   

Zhou and Bowie identified a thermostable DgkA mutant (I53C/I70L/M96L/V107D; CLLD, previously referred to as Δ4 DgkA; Li, Lyons *et al.*, 2013 ▶) upon screening a random-mutation library (Zhou & Bowie, 2000 ▶). CLLD has a half-life of 35 min in octylglucoside micelles at 80°C (Zhou & Bowie, 2000 ▶) and 3 h in decylmaltoside at 95°C (Li, Lyons *et al.*, 2013 ▶). We found that crystals obtained using this mutant diffracted to a higher resolution when compared with crystals of wild-type (WT) DgkA (Li, Lyons *et al.*, 2013 ▶; Li, Shah *et al.*, 2013 ▶). Accordingly, the assorted constructs reported here originated either directly or indirectly from CLLD. All mutations were generated using PCR-based site-directed mutagenesis. The identity of the cloned genes was verified by DNA sequencing (MWG Biotech). Recombinant proteins were produced and purified as described previously (Li & Caffrey, 2011 ▶; Li, Lyons *et al.*, 2013 ▶; Li, Shah *et al.*, 2013 ▶).

For SeMet labelling, the pTrcHisB vector carrying the desired *dgkA* mutant was transformed into the methionine-auxotroph *Escherichia coli* strain B834 (DE3) (Novagen). A single colony was used to inoculate 20 ml Luria–Bertani (LB) broth supplemented with 100 mg l^−1^ ampicillin, and the cells were allowed to grow overnight at 37°C with shaking at 200 rev min^−1^. Following centrifugation at 1000*g* for 3 min, the cell pellet was resuspended in 50 ml M9 minimal medium [without Met; 1.28%(*w*/*v*) Na_2_HPO_4_.7H_2_O, 0.3%(*w*/*v*) KH_2_PO_4_, 0.25%(*w*/*v*) NaCl, 0.5%(*w*/*v*) NH_4_Cl, 2 m*M* MgSO_4_, 0.1 m*M* CaCl_2_, 0.5%(*w*/*v*) glucose, 2 mg l^−1^ thiamine, 2 mg l^−1^ biotin, 40 mg l^−1^ of all amino acids except Met and 100 mg l^−1^ ampicillin] before being seeded into 2 l M9 minimal medium without Met. The culture was allowed to grow at 37°C for 30 min in a shaking incubator at 200 rev min^−1^ to consume the Met carried over from the LB medium. SeMet (catalogue No. S3132, Sigma) was then added to a final concentration of 40 mg l^−1^. The cells were induced with 1 m*M* isopropyl β-d-1-thiogalactopyranoside (IPTG) at an OD_600_ of 0.6–0.8 for 6 h. The biomass was harvested and DgkA purification was carried out as described previously (Li & Caffrey, 2011 ▶; Li, Lyons *et al.*, 2013 ▶; Li, Shah *et al.*, 2013 ▶).

Kinase activity assays were performed with the protein reconstituted in the cubic phase using published procedures (Li & Caffrey, 2011 ▶). Details regarding *in meso* crystallization and crystal harvesting have been reported in Li, Boland, Aragao *et al.* (2012 ▶).

Stock solutions of heavy atoms were generally made at 10 m*M* in Milli-Q water. Because of low solubility, mersalyl acid (C_13_H_18_HgNO_6_), thiomersal (C_9_H_9_HgNaO_2_S) and tantalum bromide (Ta_6_Br_12_) were made at 0.5, 0.5 and 0.7 m*M*, respectively, in the relevant precipitant solution. Toxic chemicals were weighed using a dedicated balance inside a fume hood. The heavy atoms used in this study are summarized in Table 1 ▶. The concentration of the Ta_6_Br_12_ solution was determined in a plate reader (Molecular Devices M2^e^) using a molar extinction coefficient of 6600 *M* ^−1^ cm^−1^ (Vogler & Kunkely, 1984 ▶).

#### 2.2.2. Heavy-atom co-crystallization in the lipid mesophase   

For co-crystallization, heavy atoms were added to the precipitant screen solutions to the desired concentration (Table 1 ▶). The precipitant solutions for 7.8 MAG and 9.9 MAG were 7.8%(*v*/*v*) MPD, 100 m*M* NaCl, 100 m*M* LiNO_3_, 100 m*M* sodium citrate pH 5.6 and 5.5%(*v*/*v*) 2-methyl-2,4-pentanediol (MPD), 100 m*M* NaCl, 100 m*M* LiNO_3_, 60 m*M* magnesium acetate, 50 m*M* sodium citrate pH 5.6, respectively. Trials were set up using a Mosquito LCP robot (TTP Labtech) as described in Li, Boland, Walsh *et al.* (2012 ▶). This instrument uses disposable tips, which is a real advantage in that contamination of the instrument with heavy atoms is minimized. Each well in the crystallization plate contained 50 nl lipid mesophase covered with 800 nl heavy-atom-containing precipitant solution.

#### 2.2.3. Heavy-atom soaking of crystals grown in the lipid mesophase   

**Figure 1:**
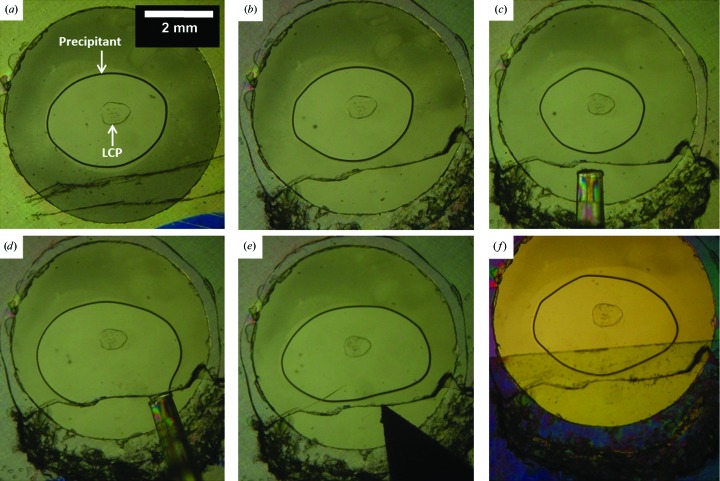
Procedure for heavy-atom soaking in 96-well glass sandwich plates. A typical well for lipid cubic phase crystallization is shown in (*a*). 50 nl lipid cubic phase (LCP) is covered with 800 nl precipitant solution in a glass sandwich plate. For soaking, wells identified for use in heavy-atom soaking experiments were cut with a glass cutter (*a*) to create a window (*b*) for injection of 800 nl heavy-atom solution (*c*, *d*) into the wells. The cover slide was raised slightly using the tip of the glass cutter (*e*) to avoid contact between the heavy-atom solution and the glass edge during soaking. The well was sealed with 3 × 7 mm strips of tape (*f*) to prevent evaporation during soaking. To back-soak, the tape was removed, the heavy-atom solution was wicked away with tissue paper and heavy-atom-free precipitant solution was added, repeating steps (*c*)–(*f*).

Heavy-atom soaking of crystals grown in the lipid mesophase was carried out as illustrated in Fig. 1 ▶. After soaking for the desired time (hours to days) at 4°C (crystals grew at 4°C; soaking should be performed at the crystallization temperature), the heavy-atom solution was replaced with precipitant solution to back-soak. Crystals were harvested as described in Li, Boland, Aragao *et al.* (2012 ▶), taking particular care to avoid contact with heavy atoms. All procedures were performed using safety glasses and two pairs of protective gloves.

#### 2.2.4. Cysteine-accessibility assay using Ellman’s reagent (DTNB)   

The principle of the assay is described in §[Sec sec3.6]3.6. The protein was solubilized in buffer *A* consisting of 1 m*M* tris(2-carboxyethyl)phosphine (TCEP), 0.25%(*w*/*v*) *n*-decyl-β-d-maltopyranoside (DM), 0.1 *M* NaCl, 10 m*M* Tris–HCl pH 7.8. Because the reducing agent, TCEP, reacts with DTNB (Shafer *et al.*, 2000 ▶), it was removed by washing the Ni–NTA-bound protein with TCEP-free buffer *A*. Eluted protein at 1 mg ml^−1^ was incubated with test mercury compounds at a 1:3 molar ratio of protein:mercury at room temperature (RT, 20–21°C) for 30 min. To initiate the assay, DTNB (1.2 µl of 33 m*M* DTNB stock in sodium phosphate buffer pH 8.0) was added to 200 µl protein solution in a 96-well plate (catalogue No. 265301, Nunc). Controls were set up using protein without mercury treatment and buffer without protein. The protein concentration was 30 µ*M* in 0.25%(*w*/*v*) DM, 100 m*M* NaCl, 10 m*M* Tris–HCl pH 7.8. The reaction was allowed to proceed for 1 h at 30°C inside the microplate reader (M2^e^, Molecular Devices). The absorbance at 412 nm was recorded every 2 min.

#### 2.2.5. Pre-labelling of single-Cys mutants for crystallization   

30 µl of labelling solution consisting of 12 mg ml^−1^ DgkA (0.8 m*M*) and 2.4 m*M* mercury compound [HgCl_2_, Hg(O_2_CCH_3_)_2_ (mercury acetate), CH_3_HgCl or C_2_H_5_HgOH_2_PO_3_ (ethylmercury phosphate; EMP)] were incubated at RT for 30 min. The volume of mercury compound-containing solution added was very small (0.7 µl from 0.1 *M *stock) in order to minimize the change in protein concentration. Excess free mercury compound was removed by dialysis in a cassette fashioned from an Eppendorf tube as follows. The protein–mercury solution was placed in the upturned lid (detached from the tube) of a 1.5 ml Eppendorf tube and was covered with a layer of dialysis membrane (molecular-weight cutoff 14 kDa). The upper ∼1 cm of the tube was cut from the rest of the tube and placed on the lid over the dialysis membrane to create a tight-fitting seal. This simple dialysis button was placed with the dialysis membrane face down in a beaker containing 200 ml dialysis buffer [0.25%(*w*/*v*) DM, 100 m*M* NaCl, 1 m*M* TCEP, 10 m*M* Tris pH 7.8]. Dialysis was allowed to proceed at RT overnight and for an additional 6 h with 200 ml freshly prepared dialysis buffer the next day. Protein solution was recovered by puncturing the dialysis membrane with a pipette tip followed by aspiration. Parenthetically, as an alternative to the home-made buttons just described, a 96-well micro-dialysis plate could be used (Thermo Scientific Pierce). The protein-laden cubic phase prepared with mercury-labelled kinase and 7.8 MAG was used to set up crystallization trials as described in §2.2.1 (Li, Shah *et al.*, 2013 ▶).

#### 2.2.6. Mass spectrometry (MS) analysis   

DgkA protein (0.1 mg in ∼0.4 ml reaction mixture; §[Sec sec2.2.4]2.2.4) was precipitated at RT by adding 0.4 ml 30%(*w*/*v*) trichloroacetic acid. After washing the precipitate three times with 1 ml Milli-Q water, the air-dried (overnight at RT) sample was sent to the Astbury Centre for Structural Molecular Biology, University of Leeds, England for electrospray ionization mass spectrometry (ESI-MS) using a carrier solution containing 50%(*v*/*v*) acetonitrile and 0.1%(*v*/*v*) formic acid.

#### 2.2.7. Diffraction data collection   

Diffraction data were collected on GM/CA-CAT beamline 23-ID-B at the Advanced Photon Source (APS) and on beamline I24 at Diamond Light Source (DLS). At the APS, data were recorded using a MAR 300 CCD detector with 1° oscillation and 1 s exposure per image, a collimated beam size of 10 µm and a sample-to-detector distance of 350–500 mm. At the DLS, data were recorded on a PILATUS 6M detector with 0.2° oscillation and 0.2 s exposure per image, a micro-focus beam size of 10 µm and a sample-to-detector distance of 500–650 mm. Fluorescence scans around the chosen absorption edge were performed at the beamline on snap-cooled crystals in loops using the automated procedures implemented at the beamline. Primarily, diffraction data were recorded at the wavelength of the absorption peak (as determined by fluorescence) for each heavy atom in order to maximize the anomalous signal. Diffraction data were indexed, scaled, merged and analysed using either *XDS* and *XSCALE* (Kabsch, 2010 ▶) *via* *xia*2 (Winter *et al.*, 2013 ▶) or *MOSFLM* (Leslie, 2006 ▶), *SCALA* (Evans, 2011 ▶) and *phenix.xtriage* (Adams *et al.*, 2010 ▶). The *SHARP*/*autoSHARP* (Bricogne *et al.*, 2003 ▶; Vonrhein *et al.*, 2007 ▶), *SHELXC*/*D*/*E* (Sheldrick, 2010 ▶) and *phenix.autosol* (Adams *et al.*, 2010 ▶) software suites were used for phasing attempts.

## 3. Results and discussion   

**Figure 2:**
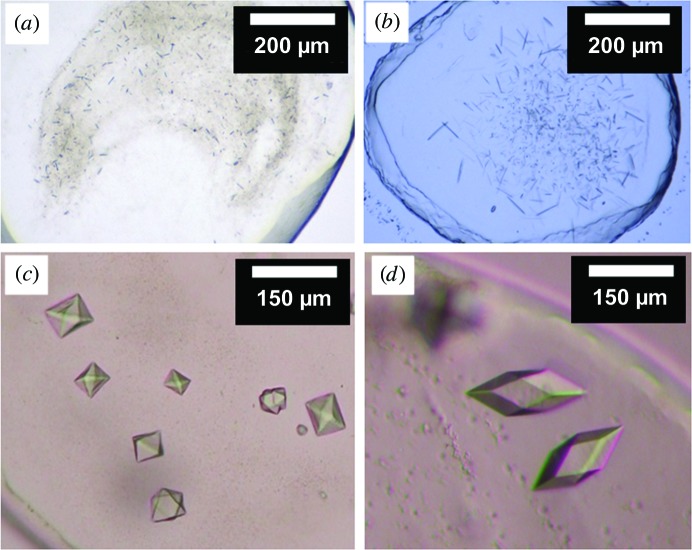
Optimizing *in meso* crystallization of DgkA by varying the temperature, salt additives and host lipid. Details of how the optimizations were performed have been reported in Li, Shah *et al.* (2013 ▶). (*a*) CLLD crystals grown at 20°C with 9.9 MAG as the host lipid and a precipitant consisting of 7.8%(*v*/*v*) MPD, 100 m*M* NaCl, 100 m*M* sodium citrate pH 5.6. (*b*) The same conditions as in (*a*) with crystallization at 4°C. (*c*) Nitrate was key to producing three-dimensional bipyramid-shaped crystals. The conditions are the same as in (*b*) with the inclusion of 100 m*M* LiNO_3_ in the precipitant solution. (*d*) CLLD DgkA crystals grown at 4°C with 7.8 MAG and a precipitant consisting of 5%(*v*/*v*) MPD, 100 m*M* NaCl, 100 m*M* LiNO_3_, 60 m*M* magnesium acetate, 50 m*M* sodium citrate pH 5.6.

The initial crystals of WT and CLLD DgkA diffracted to maximum resolutions of 4 and 3.7 Å, respectively (Li, Shah *et al.*, 2013 ▶). Subsequent optimization (Fig. 2 ▶) resulted in a 3.1 Å resolution data set for CLLD. A second crystal form obtained using a different DgkA construct (CM41, detailed in §[Sec sec3.4]3.4, previously referred to as Δ7 DgkA; Li, Lyons *et al.*, 2013 ▶) provided a 2.05 Å resolution native data set. It was with this crystal form that the structure was eventually solved (Li, Lyons *et al.*, 2013 ▶).

In this study, we describe the steps taken to go from initial crystals diffracting to ∼3.0 Å resolution to the final phased and solved structure at 2.05 Å resolution. The volume of work undertaken was immense. Like all heavy-atom derivatization studies, success can only be evaluated properly by analysing the relevant X-ray diffraction data. This makes the process extremely time-consuming, requiring rounds of protein labelling pre- or post-crystallization, crystal harvesting and shipping to a synchrotron source, diffraction data collection and data analysis and evaluation. The time between performing an experiment to test a particular condition or treatment and having a result with which to redirect the project occasionally extended to many months.

In the case of *in meso*-grown crystals the situation was additionally challenging for several reasons. At the time the work was being performed, only two synchrotron beamlines in the world (GM/CA-CAT 23-ID-B/D at APS, USA and I24 at DLS, England) were suitable for X-ray data collection from *in meso*-grown crystals. This was because a micrometre-sized beam with rastering capabilities was needed to locate, centre and collect data from crystals of maximum dimension 50–70 µm that were usually buried and invisible in an opaque, snap-cooled mesophase (Cherezov *et al.*, 2009 ▶). Of course, access to such beamlines was, and still is, limited owing to oversubscription. Furthermore, the actual process of finding and centring crystals by diffraction rastering was then quite time-consuming. A single crystal could only be evaluated and data collected, if suitable, every 15 min or so. An additional complication arose owing to the fact that the lipid, 7.8 MAG, used to create the hosting mesophase for crystal growth was not available commercially at the time and had to be produced in-house using procedures that are time-consuming and expensive. Typically, it takes a skilled synthetic organic chemist three weeks to synthesize and to purify approximately 2 g of lipid. This does not include the time required to perform the necessary quality-control tests.

We next describe the assorted strategies and approaches taken to phase and to solve the structure of DgkA. The story is told with a view to informing and enlightening others considering embarking on such an endeavour that can be fraught with difficulties but that will hopefully lead to a satisfying result in the form of a high-resolution structure of a high-impact integral membrane protein.

### 3.1. Initial attempts at phasing using SeMet-labelled CLLD   

**Figure 3:**
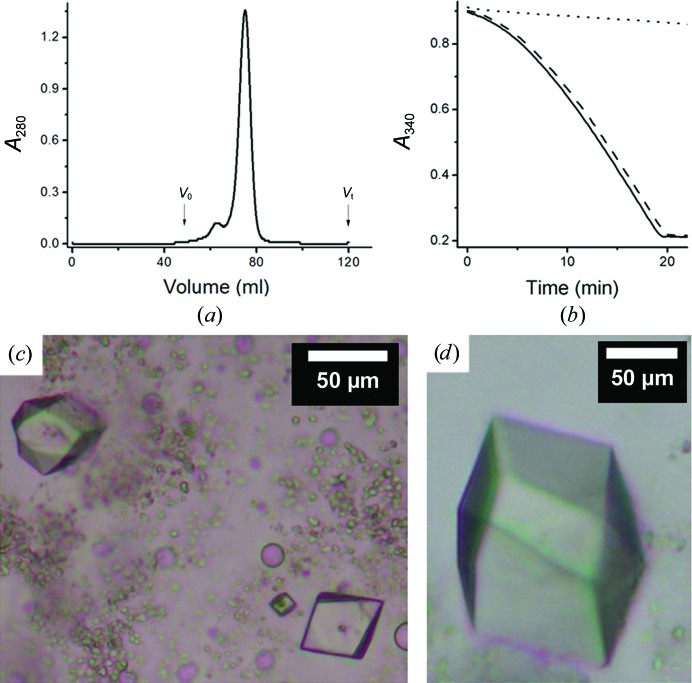
Characterization of SeMet-labelled CLLD DgkA. (*a*) Gel-filtration profile of SeMet CLLD. *V*_0_ and *V*_t_ indicate the void and total volume of the column, respectively. The elution volume for the protein is 74.9 ml. (*b*) Progress curve of the coupled assay reaction in the absence of protein (dotted line) and in the presence of CLLD (dashed line) and SeMet CLLD (solid line). Monoolein was used as the host lipid as well as the lipid substrate for the *in meso* assay of DgkA. (*c*) *In meso* crystals of SeMet CLLD grown at 4°C with 9.9 MAG and a precipitant solution consisting of 8.2%(*v*/*v*) MPD, 4%(*v*/*v*) 1,4-butanediol, 100 m*M* NaCl, 100 m*M* LiNO_3_, 100 m*M* sodium citrate pH 5.6. (*d*) *In meso* crystals of SeMet CLLD grown at 4°C with 7.8 MAG as the host lipid and a precipitant solution consisting of 4.5%(*v*/*v*) MPD, 100 m*M* NaCl, 60 m*M* magnesium acetate, 100 m*M* LiNO_3_, 50 m*M* sodium citrate pH 5.6.

As noted, the initial crystals of DgkA diffracted at best to 3.1 Å resolution (Li, Shah *et al.*, 2013 ▶). Our first attempt at phasing was by SAD/MAD using SeMet-labelled protein. The protein has 121 residues, two of which are methionines, which was expected to be sufficient should the protein be successfully expressed with both as SeMet. The yield of SeMet-labelled CLLD was 5 mg per litre of culture, which is about half of that obtained with unlabelled protein. This was not unexpected because the biomass produced on M9 minimal medium is generally less than that from LB medium. The labelled protein eluted as a Gaussian-shaped peak at the expected elution volume on a size-exclusion chromatographic column without significant aggregation (Fig. 3 ▶ *a*). The kinase activity of the protein when reconstituted into the bilayer of the cubic phase was 17.0 µmol mg^−1^ min^−1^ (Fig. 3 ▶ *b*), as observed with unlabelled CLLD (Li & Caffrey, 2011 ▶; Li, Shah *et al.*, 2013 ▶; Li, Lyons *et al.*, 2013 ▶). These data suggested that the assumed replacement of sulfur with selenium at Met63 and Met66 did not alter the structure or the enzymatic activity of the protein and that it was suitable for use in crystallization trials. The SeMet-labelled protein crystallized readily in both 7.8 MAG and 9.9 MAG (Figs. 3 ▶ *c* and 3 ▶ *d*). However, the crystals proved to be particularly sensitive to radiation damage, and only diffracted to a maximum of 5 Å resolution on the GM/CA-CAT beamline despite extensive screening. Because of the poor resolution, the SeMet approach to phasing with CLLD was not pursued further.

### 3.2. Co-crystallization with heavy atoms in the lipid mesophase   

Because the SeMet-labelled CLLD crystals did not diffract well and were radiation sensitive, heavy atoms other than selenium and indeed entirely different labelling strategies needed to be considered. Heavy-atom labelling is usually performed using one of the following methods.

(i) Co-crystallization. In this method, the heavy atom is present in the precipitant solution at the time that the crystallization trial is set up. Accordingly, the heavy atom is expected to bind to the protein prior to and/or during crystal growth.
(ii) Soaking. Here, the heavy atom is added to label extant crystals.
(iii) Pre-labelling. Following this method, the protein is labelled, for example *via* mercury covalently bound to, ideally, site-selected cysteine residues (Martinez *et al.*, 1993 ▶), and then used to set up the crystallization trial.

Co-crystallization is used less often because of the potential for non-isomorphism between crystals of labelled and non­labelled protein. However, this approach does have the advantage that it can be performed with higher throughput than the soaking method. Typically, the precipitant solution is doped with different heavy atoms across a range of concentrations in a multi-well format. The subsequent steps of setting up crystallization trials, monitoring for crystal growth and harvesting are no different than for the native variant. Therefore, this approach was tried next with DgkA.

**Figure 4:**
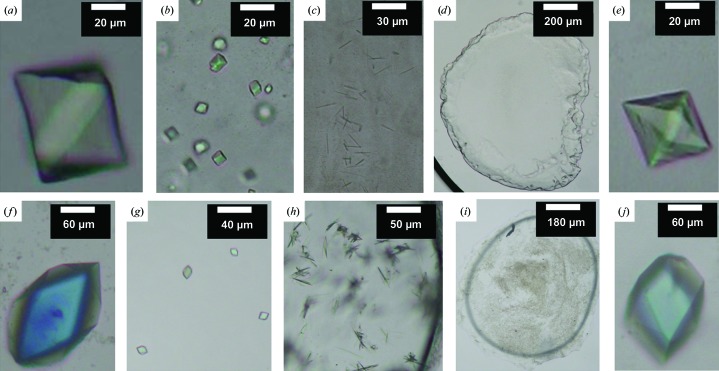
Representative results for heavy-atom co-crystallization trials. Trials were carried out using CLLD with 9.9 MAG (*a*–*e*) and 7.8 MAG (*f*–*j*) as host lipid at 4°C. The basic condition (without heavy atom) consisted of 7.8%(*v*/*v*) MPD, 100 m*M* NaCl, 100 m*M* LiNO_3_, 100 m*M* sodium citrate pH 5.6 for 9.9 MAG and of 5%(*v*/*v*) MPD, 100 m*M* NaCl, 100 m*M* LiNO_3_ 60 m*M* magnesium acetate, 50 m*M* sodium citrate pH 5.6 for 7.8 MAG. The conditions with heavy atoms were as follows: (*a*) no heavy atom, (*b*) 0.1 m*M* mersalyl acid, (*c*) 0.1 m*M* K_2_PtCl_4_, (*d*) 0.5 m*M* NaAuCl_4_, (*e*) 0.5 m*M* Sm(NO_3_)_3_, (*f*) no heavy atom, (*g*) GdCl_3_, (*h*) 0.2 m*M* HgCl_2_, (*i*) 0.1 m*M* K_2_PtCl_4_ and (*j*) 0.5 m*M* Sm(NO_3_)_3_. Images (*a*)–(*e*) and (*g*)–(*i*) were recorded with normal light; images (*f*) and (*j*) were recorded between crossed polarizers.

Crystallization trials involving CLLD were carried out with and without heavy atoms in the precipitant solution (Table 1 ▶), first using 9.9 MAG as the host lipid. In the absence of heavy atom, the crystals are bipyramid-shaped (Fig. 4 ▶ *a*). The crystal shape remained the same in the presence of most heavy atoms tested at low concentrations (40–100 µ*M*), but the crystals that formed were generally a lot smaller than those grown without added heavy atoms (Fig. 4 ▶ *b*). At 0.2 m*M* or higher, in most cases the crystals either had a different morphology (Fig. 4 ▶ *c*) or failed to grow (Fig. 4 ▶ *d*). Exceptions were noted with Sm(NO_3_)_3_ and Pb(NO_3_)_2_, where the crystals retained the original shape up to 0.5 m*M* (Fig. 4 ▶ *e*). This same experiment was repeated with 7.8 MAG as the host lipid, because crystals grown in 7.8 MAG were larger and diffracted better than those grown in 9.9 MAG (Fig. 2 ▶). Similar results were observed (Figs. 4 ▶ *f*–4 ▶ *j*). Again, Sm(NO_3_)_3_ and Pb(NO_3_)_2_ had little effect on CLLD crystal morphology up to 0.5 m*M*.

The small (2–10 µm) crystals grown at low concentrations of heavy atom (Figs. 4 ▶ *b* and 4 ▶ *g*) were not tested for diffraction quality because of the time involved in arranging for and making such measurements and the anticipated extreme sensitivity to radiation damage owing to the diminutive crystal size and the presence of heavy atoms. Crystals with morphologies that differed with respect to those observed with nonlabelled protein were not tested either because they were typically very thin needles that are often difficult to harvest and diffract weakly (Figs. 4 ▶ *c* and 4 ▶ *h*). Crystals obtained in the presence of Sm and Pb in 7.8 MAG and 9.9 MAG were tested. Most diffracted to 5–6 Å resolution, while a few data sets ranged from 3.7 to 4.3 Å resolution. Upon reduction and analysis of the diffraction data using either *XDS* and *XSCALE* (Kabsch, 2010 ▶) *via* *xia*2 (Winter *et al.*, 2013 ▶) or *MOSFLM* (Leslie, 2006 ▶), *SCALA* (Evans, 2011 ▶) and *phenix.xtriage* (Adams *et al.*, 2010 ▶), there was very limited anomalous signal at very low resolution (SIGANO < 1 at resolutions higher than 8 Å) at best, and certainly not enough to phase the structure.

We had hoped to use co-crystallization to screen for potential binders for use in soaking experiments based on the following logic: because the heavy atoms in the co-crystallization trials were used at low (micromolar) concentrations, their effect on crystallization (preventing crystallization, altering morphology *etc.*) would be likely to be result of the heavy atom interacting directly with the protein and not as a result of changing the physico-chemical properties of the precipitant such as the ionic strength, dielectric constant and so on. Therefore, the change in crystal morphology, if it did occur, could be used as a reasonably reliable criterion to identify binders for use in subsequent soaking experiments. However, as noted, almost all of the heavy atoms tested, 48 in total, affected the crystallization behaviour of CLLD. Therefore, the co-crystallization study did not prove useful as a screen to aid in the identification of a few select heavy atoms with which to proceed to diffraction measurements and/or to soaking experiments.

### 3.3. Soaking heavy atoms into crystals in the lipid mesophase   

Protein crystals grow in a mesophase that is sandwiched between glass plates (Caffrey & Cherezov, 2009 ▶). This makes accessing crystals for soaking a real challenge. Because of the large number of soakings expected in this study, alternatives to the default glass sandwich plate that might facilitate the process were investigated. To begin with, materials other than glass were examined. In one instance, ClearSeal (Hampton Research HR4-521), a thin plastic film, was tested because it should be easy to cut to access the crystal-laden mesophase for soaking. While the film was indeed easy to cut, an additional complication arose owing to the nature of the mesophase post-crystal growth. The CLLD crystals grew in a precipitant that includes MPD. This diol, under crystal-growth conditions, triggers a transition from the viscous cubic phase to the much more fluid sponge phase (Cherezov *et al.*, 2006 ▶). It is from the sponge phase that crystals must be harvested. With the thin ClearSeal film, as soon as it was cut, the capillarity was strong enough to deform it, which allowed the mesophase, along with its cargo of crystals and surrounding precipitant, to move and to make contact with the wall of the well. When this happens, the crystals are irretrievably lost (Li, Boland, Aragao *et al.*, 2012 ▶). Thick plastic plates were tried next, with the expectation that their rigidity would work against capillarity and loss of crystals, as just noted. However, it was not much easier to cut than glass. In addition, unlike glass, most plastics are not water-tight and, regardless of thickness, lose water over time. This creates problems in obtaining crystals reproducibly. Another approach was to use grease to coat the glue on the double-stick spacer, so that the cover glass could be removed with relative ease. To this end, the spacer was first stuck to the base plate as in the normal setup (Caffrey & Cherezov, 2009 ▶) and the protective cover was removed to expose the upper sticky surface of the spacer. This was then coated with a layer of Vaseline. Crystallization plates were filled with mesophase and precipitant, as per usual, two columns at a time. Wells were sealed with 18 × 18 × 0.2 mm glass cover slips. In this setup, the wells could be opened/closed by lifting/lowering the cover glass with ease, and thus were deemed suitable for use in soaking experiments. However, for unknown reasons the reproducibility of obtaining large CLLD crystals was very poor with this arrangement. Of course, for soaking experiments a large number of wells with large, high-quality crystals is needed. Therefore, we decided to continue the phasing quest with the standard glass sandwich plates.

The procedure used for setting up heavy-atom soaking trials in glass plates is illustrated in Fig. 1 ▶. All soaking experiments were performed at 4°C, which is the optimum temperature for DgkA crystal growth. The success rate (the number of wells from which soaked crystals were successfully harvested in relation to the total number of wells targeted) was depressingly low at 5–10% despite the fact that the work was performed by the first author, having a very skilled pair of hands and a total commitment to the project. A number of challenges were encountered along the way that contributed to the low success rate. Firstly, upon scribing with the glass cutter the cover glass can crack and break over the well, leading to loss of mesophase and crystals. Secondly, glass shards and powder created during the scribing and cutting can contribute to the loss of mesophase and crystals. Before injecting the heavy-atom solution, it is important to remove all such shards and powder at the edge of the window in the cover glass. However, this is not always a trivial task because the shards can become trapped in the 140 µm space between the glass plates, one of which is thin and easily broken. Further, upon opening the well it is very important not to leave it exposed to air for long or the mesophase and precipitant will dry out and the crystals will be lost. This means that clearing the shards should be performed as expeditiously as possible. If these are not removed, the heavy-atom solution can adsorb into the shards by capillarity as it is being added to the well. This then contacts the sealing tape, which draws all of the solution towards the tape and away from the mesophase. Glass shards can also facilitate the heavy-atom solution spreading over the well and away from the mesophase to eventually make contact with the wall of the well and not the mesophase. Thirdly, the cover glass over the well can break as it is being raised, as illustrated in Fig. 1 ▶(*e*). Fourthly, the mesophase can be lost when tape is used to seal the well. When the tape is applied to cover the open window, direct contact between the bathing solution and the tape can lead to drying, especially in the case of lengthy soaking and back-soaking experiments. Finally, harvesting crystals from wells that have had contact with heavy-atom solutions is particularly challenging because of the need for the safe handling and manipulation of these hazardous materials. This calls for the use of two pairs of gloves and safety glasses, which compromise the manual dexterity much in need for harvesting small, fragile crystals from a viscous and sticky mesophase. When all of this must be performed at 4°C, one can appreciate why the success rate is so low. It is because the success rate was so low that typically 20–30 96-well plates were set up every two weeks to ensure that sufficient crystals were available for the planned soaking experiments.

Because soaking crystals *in meso* is technically challenging, time-consuming and inefficient, it was important to limit the number of trials conducted to a few well chosen heavy-atom types. A survey of the literature identified seven compounds [HgCl_2_, K_2_HgI_4_, *p*-chloromercuribenzoic acid (PCMB), K_2_PtCl_4_, KAu(CN)_2_, UO_2_(O_2_CCH3)_2_ (uranyl acetate) and K_3_UO_2_F (tripotassium uranyl fluoride)] as highly successful for soaking experiments (Garman & Murray, 2003 ▶). A similar panel of heavy atoms for use with membrane proteins has been described (Morth *et al.*, 2006 ▶; Parker & Newstead, 2013 ▶). A few from this list were chosen for use in the current study, which included HgCl_2_, EMP, PCMB, K_2_PtCl_4_, trimethyllead acetate (TMLA) and Ta_6_Br_12_ (Table 1 ▶). Mersalyl acid was selected for its slow reactivity rate, while the lanthanide GdCl_3_ was chosen because of its strong anomalous signal (Girard *et al.*, 2002 ▶). Sm(NO_3_)_3_ and Pb(NO_3_)_2_ were included in the study because they had shown some potential in the co-crystallization experiments (§[Sec sec3.2]3.2). Combined, these provided a broad range of heavy-atom characteristics with regard to partitioning coefficient (TMLA and EMP partition well into hydrophobic environments) and chemistry (Hg and Pt, covalent binding; Sm and Pb, electrostatic interaction).

**Figure 5:**
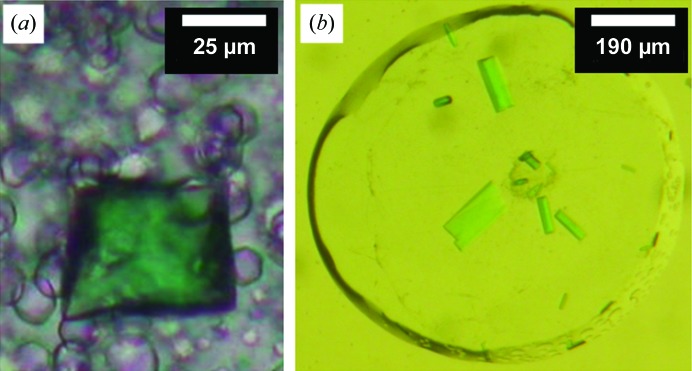
Typical results of heavy-atom soaking in the lipid mesophase. Crystals of CLLD DgkA in 9.9 MAG (*a*) and CM41 in 7.8 MAG (*b*) incubated with 0.7 m*M* Ta_6_Br_12_ (a green-coloured compound). The effectiveness of soaking is clearly visible by the green staining of the crystals. The crystals were soaked for 7 h followed by 2 h of back-soaking. The precipitant solution consisted of 8.3%(*v*/*v*) MPD, 2.4%(*v*/*v*) 1,4-butanediol, 100 m*M* NaCl, 100 mM LiNO_3_, 100 m*M* sodium citrate pH 5.6 in (*a*) and 5%(*v*/*v*) MPD, 100 m*M* NaCl, 60 m*M* magnesium acetate, 50 m*M* sodium citrate pH 5.6 in (*b*).

Despite the challenges, the soaking trial went well. For the most part, the cubic phase appeared to be unaffected by soaking and the heavy atoms diffused into the mesophase, as nicely illustrated when the bright green Ta_6_Br_12_ was used (Fig. 5 ▶). It took about 40 min for this water-soluble cluster (molecular weight 2044.5 Da) to diffuse throughout the 50 nl (0.14 mm height, ∼0.65 mm diameter) of mesophase at 4°C, as judged by the uniformity of the colour in the bolus. With this as a marker, all soaking treatments that involved water-soluble compounds lasted at least 1 h. Mersalyl acid is relatively hydrophobic and will partition, to some degree, into the bilayer of the mesophase, in which its diffusion rate drops. In this case the soaking time was increased to at least 6 h based on prior knowledge regarding the transport of hydrophobic substances in the cubic phase (Caffrey, 2009 ▶). Generally, back-soaking was performed with the aim of removing nonspecifically bound and free heavy atoms to reduce background.

DgkA crystals proved quite resistant to heavy-atom treatment in the sense that they did not dissolve or lose birefringence upon soaking, even at high concentrations (25 m*M* K_2_PtCl_4_ and 0.1 *M* TMLA, for example). Unfortunately, however, the crystals usually showed poor and anisotropic diffraction with reflections to 5 Å resolution and complete data sets to no better than 7 Å resolution. Further, the reflections were often streaky, indicating high mosaicity. A few data sets ranging from 3.5 to 4.2 Å resolution were obtained with mersalyl acid-treated crystals. Data sets for Ta_6_Br_12_-soaked crystals ranged from 3.5 to 6.7 Å resolution. The best data sets to 3.5 Å resolution were observed with HgCl_2_, K_2_PtCl_4_ and NaAuCl_4_. However, despite the reasonably good resolution, the anomalous signal was very low for the individual data sets. The radiation sensitivity of the crystals meant that collecting highly redundant data sets with a view to increasing the anomalous signal was not possible. Another problem arose in certain cases when background diffraction from the hosting mesophase was strong, leading to reduced completeness in certain resolution shells. Additionally, data sets originating from single crystals were often too non-isomorphous to be scaled together for MIR phasing. In all, the soaking approach failed to provide a route to phasing the DgkA structure.

### 3.4. Rational design of a Cys-less DgkA template for generating single-cysteine mutants   

As it did not appear likely that we could solve the structure using heavy-atom derivatives following the procedures outlined above, it was decided to explore a method that uses engineered cysteine residues for mercury binding. The approach involves removing non-essential cysteines in the target protein and placing cysteine residues systematically at sites in the protein predicted to be accessible for labelling. Following this strategy, the mercury site is pre-determined for each mutant/construct. In addition, because the free thiol of defined and accessible cysteines can form a covalent bond with mercury, the heavy-atom occupancy is expected to be high. This approach has been applied to soluble (Nagai *et al.*, 1990 ▶; Doyle *et al.*, 1996 ▶; Nureki *et al.*, 1998 ▶) and membrane (Doyle *et al.*, 1998 ▶; Jiang *et al.*, 2002 ▶; Abramson *et al.*, 2003 ▶; Jiang *et al.*, 2003 ▶; Ujwal *et al.*, 2008 ▶; He *et al.*, 2010 ▶) proteins with considerable success. The cysteine mutants, when labelled, each have the potential of providing experimental phases by SAD/MAD, or a combination of data from different cysteine mutants could be used for MIR/MIR(AS).

**Figure 6:**
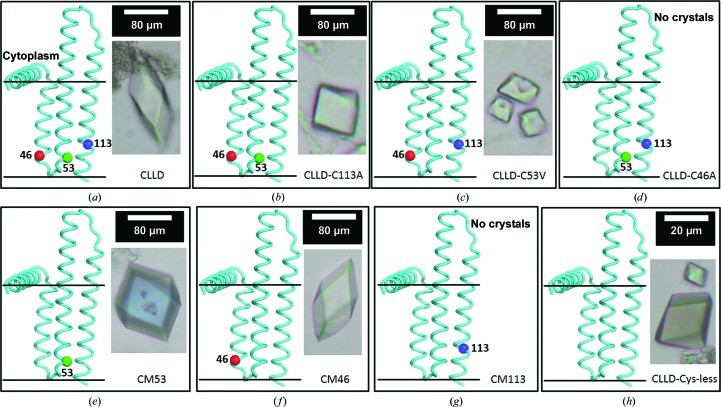
Cysteine mutants of DgkA and their crystallization behaviour. CLLD has three Cys residues (*a*). Cys46 and Cys113 are native residues, whereas Cys53 is one of the four stabilizing mutations (I53C, I70L, M96L and V107D). By removing the three Cys residues by site-directed mutagenesis, three mutants with two Cys residues (*b*–*d*), three mutants with one Cys residue (*e*–*g*) and one Cys-less mutant (*h*) were obtained. For each mutant, the crystallization behaviour is shown to the right of each panel; the location (C^α^) of the relevant Cys residue(s) in the protein is/are shown to the left of the panel. The name of each Cys mutant is indicated in the bottom right-hand corner of each panel. Precipitant solutions consisted of 4–6%(*v*/*v*) MPD, 100 m*M* NaCl, 100 m*M* LiNO_3_, 60 m*M* magnesium acetate, 50 m*M* sodium citrate pH 5.6. All trials were performed at 4°C with 7.8 MAG as the host lipid.

CLLD has cysteine residues at positions 46, 53 and 113 (Fig. 6 ▶ *a*). Cys46 and Cys113 are present in the WT enzyme, whereas Cys53 was introduced as a stabilizing mutant in the design of CLLD (Zhou & Bowie, 2000 ▶). To begin the process of selective, single-site labelling, it was desirable to have a Cys-less construct available and to use it to generate single-cysteine mutants for mercury labelling. To generate a Cys-less construct, the three cysteine residues in CLLD needed to be replaced by other amino acids. Ala was chosen for positions 46 and 113 because it had been shown that both can be replaced with Ala without compromising the enzymatic activity (Nagy *et al.*, 2001 ▶; Van Horn *et al.*, 2009 ▶). Val was chosen to replace Cys53 because Val at this position has been shown to enhance the stability of the kinase (Zhou & Bowie, 2000 ▶). By removing the three cysteine residues one, two and three at a time by site-directed mutagenesis, three double-cysteine mutants (Figs. 6 ▶ *b*, 6 ▶ *c* and 6 ▶ *d*), three single-cysteine mutants (Figs. 6 ▶ *e*, 6 ▶ *f* and 6 ▶ *g*) and the CLLD Cys-less mutant (Fig. 6 ▶ *h*) were generated. The Cys-less mutant, which differed from WT DgkA at six sites (C46A, I53V, I70L, M96L, V107D and C113A), became the template for introducing single cysteine residues at defined positions. These single-cysteine constructs now have seven mutations when compared with WT DgkA. The nomenclature used to name these single-Cys mutants takes the form CM#, where CM refers to Cys mutant and # identifies the residue number of the single cysteine in the sequence. Thus, the three single-Cys mutants identified above are henceforth referred to as CM46, CM53 and CM113.

Because all of these constructs, including double-cysteine and single-cysteine mutants, differ from CLLD in the locations of cysteine residues throughout the protein, the successful derivatization of one or more of these mutants could potentially provide useful data for phasing. To this end, and also to evaluate the effect of each mutation on the crystallization of the kinase, *in meso* crystallization trials were carried out on the seven mutants. All except CLLD C46A and CM113 crystallized in CLLD-based conditions and produced crystals similar in shape to those produced by CLLD. The sizes of the crystals for CM46 and CM53 were similar to those observed with CLLD (Figs. 6 ▶ *e* and 6 ▶ *f*). Therefore, these two single-Cys mutants were chosen for mercury labelling.

**Figure 7:**
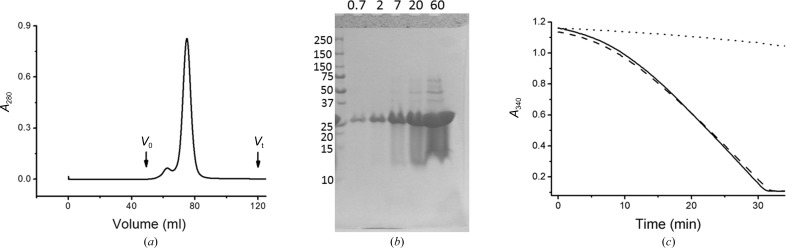
Characterization of the Cys-less CLLD mutant. (*a*) Gel-filtration profile of the mutant. *V*_0_ and *V*_t_ indicate the void and total volumes of the column, respectively. The elution volume is 74.8 ml. (*b*) Coomassie Blue-stained SDS–PAGE of the protein with the amount loaded indicated at the top of each lane in µg. Molecular-weight standards are included in the left lane (labelled in kDa). The mutant runs at ∼27 kDa, as observed for CLLD DgkA, suggesting that they share the same oligomeric state (Li, Shah *et al.*, 2013 ▶). (*c*) Progress curve of the coupled kinase reaction recorded in the absence of protein (dotted line) and in the presence of CLLD (solid line) and the Cys-less mutant (dashed line). 9.9 MAG was used as the host lipid as well as the lipid substrate for the *in meso* assay of DgkA (Li & Caffrey, 2011 ▶).

Despite their smaller size, the crystals of the Cys-less mutant displayed the same morphology as those of CLLD (Fig. 6 ▶ *h*). In addition, the yield, purity, gel-filtration and kinase-activity (16.5 µmol min^−1^ mg^−1^) characteristics of this mutant were the same as those of CLLD (Fig. 7 ▶). On the basis of SDS–PAGE analysis, just like CLLD, CLLD Cys-less also ran as a trimer (Fig. 7 ▶ *b*), suggesting that it too was thermostable, as separately verified (Li, Shah *et al.*, 2013 ▶). The latter all served as valuable characteristics of the Cys-less construct, which was to act as the template for generating single-cysteine mutants for mercury labelling.

### 3.5. Design and crystallization of single-Cys mutants   

Having established the Cys-less template for generating single-Cys mutants, our next task was to prioritize the sites in this 121-residue kinase to change to cysteine for mercury labelling. The following criteria were used to guide the process.

(i) The mutant should be enzymatically active because, at the very least, inactivity could reflect the fact that the protein is misfolded, which in turn could potentially lead to a physiologically irrelevant crystal structure.
(ii) Mutants with known defects in folding (Van Horn *et al.*, 2009 ▶) should be avoided.
(iii) For accessibility reasons, priority should be given to residues that are expected to be exposed to the aqueous environment or are near the membrane boundary in the native membrane.

As DgkA was one of the first membrane enzymes to be isolated and extensively studied biochemically and biophysically, much is known about it. Bowie and coworkers explored the tolerance of each residue in DgkA to being mutated to each of the 19 other natural amino acids (Wen *et al.*, 1996 ▶). From this work, sites suitable for making the proposed single-cysteine constructs are apparent. More directly relevant to the task at hand, Sanders and coworkers (Van Horn *et al.*, 2009 ▶) systematically mutated all residues in DgkA to cysteine. Each mutant was assayed for kinase activity, with the protein reconstituted into micelles and liposomes. The yield, folding and aggregation propensity as well as the stability characteristics of many of the mutants have also been reported (Van Horn *et al.*, 2009 ▶). Furthermore, the NMR structure solved by the Sanders group (Van Horn *et al.*, 2009 ▶), together with earlier topology models (Smith *et al.*, 1994 ▶), provided insights regarding the expected location, and thus the suitability as a site for mercury labelling, of each residue in the crystal structure.

A total of 18 single-Cys constructs were designed, with the following sites chosen for mutation: 16, 22, 23, 41, 42, 43, 46, 47, 49, 51, 53, 62, 65, 82, 86, 105, 112 and 113. 2 l of biomass were produced for each mutant using constructs made by site-directed mutagenesis. The yield of pure mutant protein ranged from 3 to 10 mg per litre of culture, which was deemed to be adequate to proceed to crystallization trials. To reduce the magnitude of the screening effort, 7.8 MAG was selected as the host lipid in which to perform trials for most of the mutants examined because CLLD crystals grown in this lipid were generally larger and better diffracting.

**Figure 8:**
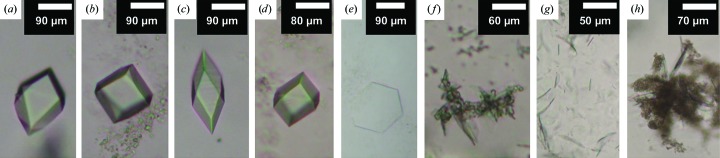
*In meso* crystallization of single-Cys mutants of DgkA: (*a*) CM41, (*b*) CM42, (*c*) CM43, (*d*, *e*) CM62, (*f*) CM47, (*g*) CM102, (*h*) CM105. The crystallization conditions are 4–6%(*v*/*v*) MPD, 50–100 m*M* NaCl, 30–60 m*M* magnesium acetate, 100–200 m*M* LiNO_3_, 50 m*M* sodium citrate pH 5.6 for (*a*)–(*d*) and (*f*)–(*h*) and 5%(*v*/*v*) MPD, 100 m*M* LiNO_3_, 100 m*M* (NH_4_)_2_HPO_4_, 30 m*M* magnesium acetate, 50 m*M* sodium citrate pH 5.6 for (*e*). Crystals were grown at 4°C with 7.8 MAG as the host lipid.

Of the 18 mutants screened, nine produced crystals and six (CM41, CM42, CM43, CM46, CM53 and CM62) grew the preferred bipyramid-shaped crystals (Figs. 8 ▶ *a*, 8 ▶ *b*, 8 ▶ *c*, 8 ▶ *d*, 6 ▶ *e* and 6 ▶ *f*), five of which were chosen for mercury labelling. CM62 was not selected because of its irreproducibility in generating bipyramid-shaped crystals.

### 3.6. Assay development to assess the reactivity of cysteine residues in DgkA mutants   

**Figure 9:**
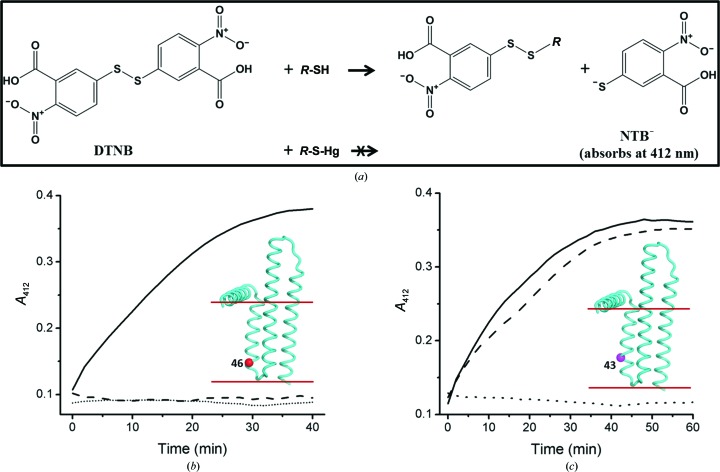
The principle of and representative results from the cysteine-accessibility assay using Ellman’s reagent. (*a*) DTNB reacts with accessible thiols such as cysteine residues in the protein to generate NTB^2−^, which absorbs at 412 nm. Mercury binds to accessible cysteine residues by forming a covalent Hg—S bond, thus preventing the production of NTB^2−^ upon the addition of DTNB. (*b*) Progress curve of DTNB reacting with CM46 with (dashed line) or without (solid line) pre-labelling with EMP. A control sample without mercury and protein is included (dotted line). Protein was used at 30 µ*M*. EMP, when present, was incubated with protein at a 3:1 molar ratio at 20°C for 30 min prior to the addition of DTNB. DTNB was added to a concentration of 0.2 m*M* to initiate the reaction. (*c*) Progress curve of the Ellmann reaction with CM43 (30 µ*M*) without added mercury (solid line) and upon incubation with EMP (dotted line) or with Hg(O_2_CCH_3_)_2_ (mercuric acetate; dashed line). An increase of 0.26 in *A*_412_ is expected for the complete reaction of a single thiol with DTNB under the conditions indicated. The insets show the location of the single cysteine residues in the solved protein.

Given the number of mutants and the number of mercury compounds available to choose from, it was not practical to evaluate, in a reasonable timeframe, all combinations based on synchrotron data collection. Accordingly, prior knowledge regarding the reactivity of single-Cys mutants with mercury was considered vital to enable expeditious pre-screening for suitable cysteine mutants and mercury compounds. For this purpose, an assay of cysteine accessibility and reactivity that made use of Ellman’s reagent (DTNB) was developed. DTNB reacts with thiols (cysteines in the case of proteins) to form a mixed disulfide and the dianion 2-nitro-5-thiobenzoate (NTB^2−^). The latter absorbs strongly at 412 nm with a molar extinction coefficient of approximately 14 000 *M* ^−1^ cm^−1^ (Caffrey & Kinsella, 1975 ▶; Riddles *et al.*, 1983 ▶). Thus, the reaction between DTNB and exposed cysteines in the protein is reflected by an increase in the *A*_412_ over time. If the cysteine residue in question is accessible to and reacts with mercury, the covalent bond thus formed will block the Ellman reaction (Fig. 9 ▶ *a*). This is the basis for the assay.

A typical result for the assay is shown in Fig. 9 ▶(*b*). In the absence of DTNB, *A*_412_ remains fixed at a background value during the course of the assay. In the presence of a protein with a single accessible cysteine, CM46 in this instance, *A*_412_ increases over time, approaching a final value of ∼0.37 after 40 min, which is as expected for a reaction that has gone to completion. This result indicates that Cys46 is accessible and therefore reacted with DTNB, consistent with a previous study (Czerski & Sanders, 2000 ▶). For CM46 that had been pre-incubated with HgCl_2_, *A*_412_ remained low and did not change with time, suggesting that Cys46 forms a covalent bond with mercury, thus blocking the Ellman reaction. This result demonstrated that Cys46 was available and thus suitable for mercury attachment.

The assay proved useful in establishing the accessibility of cysteine residues in DgkA and the reactivity of the targeted cysteine with different mercury compounds. As shown in Fig. 9 ▶(*c*), the CM43 mutant reacts with DTNB. However, the reaction was blocked by the organic mercury compound EMP but not by inorganic mercury acetate. Together with the reactivity data on CM46, these results suggest that residue 43 is located in an environment that is more hydrophobic than that of residue 46. This is consistent with the final crystal structure of DgkA (Li, Lyons *et al.*, 2013 ▶), which shows that residue 43 is deeper in the membrane than residue 46. This makes good sense in that only an organic mercury compound is likely to access such a site, as has been observed with other membrane proteins (Lebendiker & Schuldiner, 1996 ▶; Soskine *et al.*, 2002 ▶; Boado *et al.*, 2005 ▶).

The five mutants (CM41, CM42, CM43, CM46 and CM53) identified in §[Sec sec3.5]3.5 were tested using the Ellman assay. All reacted with DNTB, and the reaction was blocked upon pre-incubation with EMP. By contrast, the inorganic compounds HgCl_2_ and Hg(O_2_CCH_3_)_2_ (mercury acetate) only blocked the reaction in the case of CM46 and CM53. This is consistent with the fact that residues 46 and 53 are at the membrane boundary and are accessible to these salts, whereas the other three are more deeply buried.

A number of different methods are available to evaluate heavy-atom binding, including a gel-shift assay (Boggon & Shapiro, 2000 ▶), an SDS–PAGE assay using fluorescent probes (Chaptal *et al.*, 2010 ▶) and MS analysis (Cohen *et al.*, 2000 ▶; Sun & Hammer, 2000 ▶). The DTNB-based assay described here complements these other methods and offers an inexpensive, sensitive and convenient alternative way in which to probe the accessibility of cysteine residues for mercury binding, among other things. The results of the assay are highly informative in that site accessibility and the extent of mercury incorporation can be quantified simultaneously. In this study, we used 6 nmol protein in a reaction volume of 200 µl in a 96-well plate. With 384-well plates, the amount of protein could be reduced by a factor of three (2 nmol per sample). Under standard assay conditions, as described, the absorbance difference reading between the labelled and unlabelled single-cysteine mutant samples is 0.3, corresponding to a strong, unambiguous signal for data analysis. The assay, which requires about 2 h to perform, requires only a spectrophotometer or a plate reader, which are standard pieces of equipment in most biochemistry laboratories. Using DTNB as the probe, the assay is inexpensive. In addition, the hazardous heavy-atom solutions are all contained in disposable Eppendorf tubes and multi-well plates, thereby avoiding direct contact with and contamination of instruments. This helps to minimize heavy-atom waste, which is expensive to dispose of safely, and limits the laboratory space that must be set aside exclusively for heavy-atom use. All of these features contribute to making the assay attractive and generally useful.

### 3.7. Evidence for mercury derivitization by MS analysis   

To further characterize the single-Cys mutants with regard to labelling with DTNB and mercury, samples of CM41, CM43 and CM46 treated with DTNB and mercury compounds were analysed by ESI-MS (§[Sec sec2.2.5]2.2.6). The results are shown in Table 2 ▶. The unlabelled single-Cys mutants all had the expected molecular weights (MW), with a major peak at the calculated MW which was usually accompanied by two minor peaks corresponding to complexes with one or two detergent (decylmaltoside) molecules. Compared with the native protein, samples that were treated with DTNB all showed an increase in molecular weight of approximately 197 Da, consistent with there being an NTB adduct on the protein (Fig. 9 ▶ *a*). Under this condition unlabelled protein was not detected, consistent with the observation that the Ellman reaction had gone to completion (Figs. 9 ▶ *b* and 9 ▶ *c*). The sample treated with mercury compounds showed three major peaks with molecular weights corresponding to the native protein and protein with mercury attached at one and two sites, indicating that the protein had been singly and doubly labelled with mercury (Table 2 ▶).

The MS results in Table 2 ▶ are worthy of further comment and, for this purpose, the CM41 sample treated with EMP will be used for discussion. Firstly, based on the MS data, only 60% of the CM41 in the sample ended up labelled with mercury. This is inconsistent with the extent of reaction observed with DTNB, which was 100%. One possible explanation for the discrepancy is that the cysteine–mercury bond is labile under the extreme acidic conditions imposed on the sample prior to [15%(*w*/*v*) TCA used for precipitation] and during [0.1%(*v*/*v*) formic acid, 50%(*v*/*v*) acetonitrile] the MS experiment. Secondly, by design, CM41 contains a single cysteine and so finding some of the sample doubly labelled with mercury deserves examination. The sample was analysed by MS under denaturing conditions. Therefore, the additional molecular weight detected is likely to originate from EMP that is bound co­valently rather than electrostatically. Mercury is known to react with the thiol group of cysteine. However, it has some reactivity with the sulfur in methionine (Isab, 1989 ▶). Because there is only one cysteine in CM41, the double labelling suggests that EMP reacted with at least one of the two Met residues (Met63/66).

The results of the cysteine-accessibility and the MS analyses support the conclusion that the cysteine mutants had indeed been labelled with mercury. Thus, the five mutants (CM41/42/43/46/53) were investigated for mercury-assisted phasing of DgkA, as described next.

### 3.8. Mercury derivatization of single-Cys mutants   

**Figure 10:**
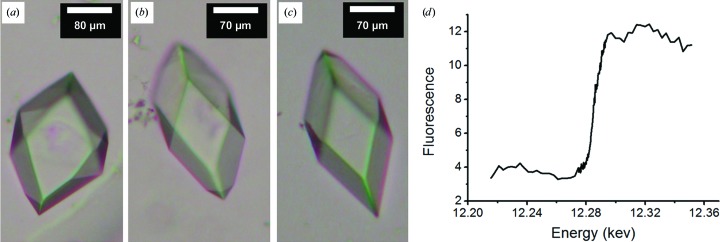
Crystals and X-ray fluorescence of single-Cys mutants of DgkA pre-labelled with mercury compounds. (*a*) CM43–EMP. (*b*) CM43–CH_3_HgCl (methylmercury). (*c*) CM46–EMP. An X-ray fluorescence scan of a crystal thus labelled indicates that mercury is present in the sample (*d*).

For mercury derivatization of the single-Cys mutants, co-crystallization (§[Sec sec2.2.2]2.2.2) with 7.8 MAG and 9.9 MAG as host lipids was investigated first because such trials are relatively easy to set up. The experiment however, resulted in either no crystal growth or needle-shaped crystals across all mutant crystals. Pre-labelling, as outlined in §[Sec sec2.2.5]2.2.5, was carried out next. The five single-Cys mutants were incubated at a protein:mercury molar ratio of 1:3. After free mercury was removed (§[Sec sec2.2.5]2.2.5), crystallization trials were set up using 7.8 MAG as the host lipid. The results are shown in Fig. 10 ▶. Bipyramidal crystals were only obtained with CM43–EMP, CM43–CH_3_HgCl (methylmercury) and CM46–EMP. The other three mutants either did not crystallize or only produced small needles following mercury pre-treatment. Finally, soaking experiments were performed by treating CM46 and CM53 crystals with HgCl_2_ and EMP (Table 1 ▶). In the case of CM41 the bipyramid crystals were less reproducible and hence were not included in the soaking study.

To test for the presence of mercury, an X-ray fluorescence scan of crystals grown from pre-labelled DgkA was performed. The spectrum (Fig. 10 ▶ *d*) has an inflection and a peak at 12.284 keV (1.0093 Å) and 12.320 keV (1.0063 Å), respectively, as expected (Hubbard *et al.*, 1994 ▶; Krishna *et al.*, 1994 ▶; Benson *et al.*, 1995 ▶; Georgiadis *et al.*, 1995 ▶; Stebbins *et al.*, 1995 ▶). This result confirms the presence of mercury in the sample.

The fluorescence signal recorded as part of the X-ray scan is, by Ockham’s razor, most likely to originate from mercury bound to DgkA in the crystal. The mercury compounds were introduced by pre-labelling the protein, which was followed by extensive dialysis to remove nonspecifically bound and free mercury (§[Sec sec2.2.5]2.2.5). Mercury was not added to the precipitant solution used for crystallization. For crystallization trials with 7.8 MAG as the host lipid, 50 nl mesophase containing 25 nl protein solution was bathed in 800 nl precipitant solution. Thus, free mercury in the cubic phase, if present at all, should be diluted 30-fold upon equilibration with the precipitant solution. All of these steps should have contributed to reducing the free mercury in the system to a negligible level. Therefore, the fluorescence signal observed was most likely to originate from mercury covalently attached to the free thiol groups in the DgkA mutants.

Unfortunately, crystals derivatized with mercury by pre-labelling or by soaking all diffracted poorly, with highest resolution reflections in the range from 6 to 9 Å.

Thus far in the quest to phase the DgkA structure, we had unsuccessfully explored experimental phasing with SeMet and heavy-atom co-crystallization, soaking and pre-labelling of ‘native’ and engineered constructs. Prospects for a solution were not encouraging. However, as an aside, we were aware of several cases in which cysteine mutants were found to diffract better than the wild-type protein (Nagai *et al.*, 1990 ▶; Doyle *et al.*, 1998 ▶; Jiang *et al.*, 2002 ▶). In our own study, we observed that the cysteine mutants also crystallized in ways that were distinctly different from the ‘native’ reference protein (Fig. 8 ▶). At this point, a decision was made to explore their potential to produce crystals of high diffraction quality where alternative routes to phasing might be possible.

### 3.9. Searching for better quality crystals   

For the purpose of optimizing crystals of over 20 mutants, a simple condition to start with is desired to reduce the number of variables to be adjusted. The most common crystallization condition and the one that produced crystals most reproducibly was chosen for this purpose. It consisted of 4–6%(*v*/*v*) MPD, 100 m*M* NaCl, 100 m*M* LiNO_3_, 60 m*M* magnesium acetate, 50 m*M* sodium citrate pH 5.6 in 7.8 MAG at 4°C. The five components in this precipitant solution had evolved over rounds of optimization (Li, Shah *et al.*, 2013 ▶). Because too many variables in a condition can complicate optimization, we first decided to identify which of the five were most critical by omitting components systematically and repeating the screening process. The view was that salt-additive identity and concentration along with pH could then be adjusted in the minimal condition for use in subsequent rounds of optimizations.

**Figure 11:**
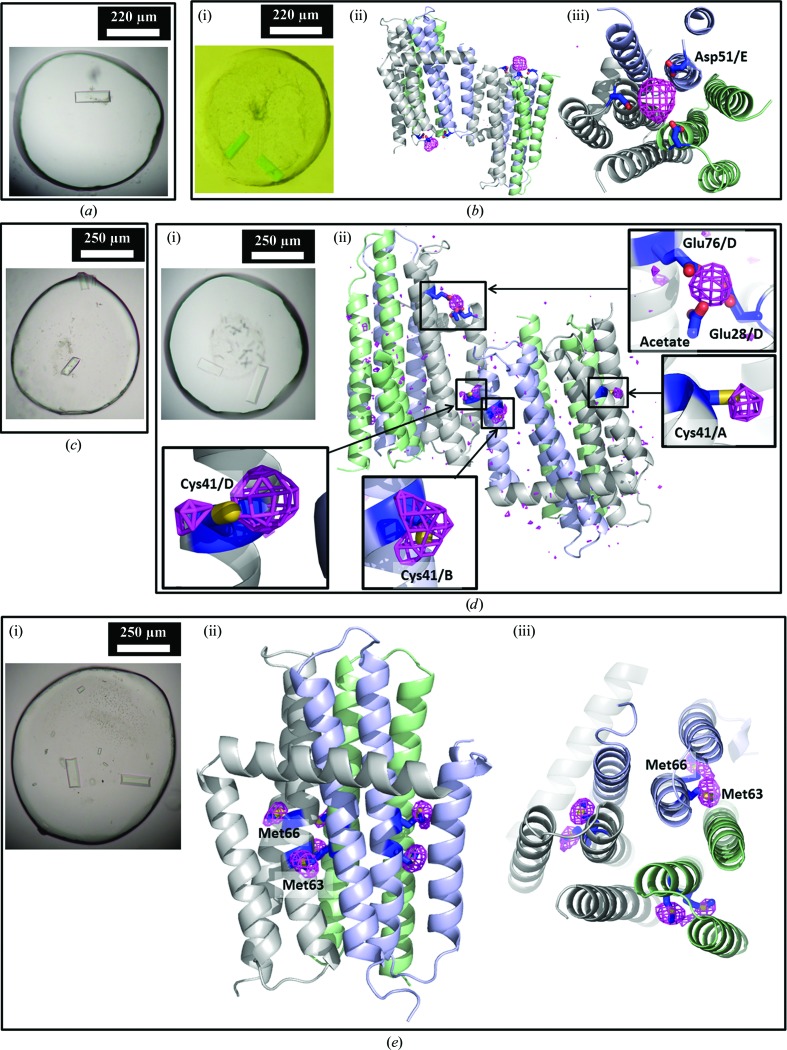
Phasing the DgkA structure using the CM41 mutant. (*a*) Native crystals of CM41. (*b*) CM41 crystals soaked with 0.7 m*M* Ta_6_Br_12_ for 7 h are shown in (i). The phased anomalous map (magenta) calculated using diffraction data collected at the tantalum edge (X-ray wavelength 1.25485 Å) and contoured at 4 r.m.s.d. shows two Ta_6_Br_12_ clusters per asymmetric unit (one per trimer) in the CM41 crystal form (ii, side view; iii, viewed from the periplasmic side). The clusters are coordinated by three Asp51 residues, one from each polypeptide in the trimer, and refined with occupancies of 40 and 50%. The protein is shown in cartoon representation with each polypeptide coloured differently. (*c*) Crystals of CM41 pre-labelled with CH_3_HgCl. (*d*) Crystals of CM41 pre-labelled with EMP are shown in (i). The anamalous map (magenta) calculated using diffraction data collected at the mercury edge (1.0063 Å) at 3 r.m.s.d. shows four mercury sites per asymmetric unit. Three are at the Cys41 position, as expected. Interestingly, anomalous signal also shows up in a region coordinated by two glutamates and acetate (ii). This site was modelled as a zinc ion in the native structure (PDB entry 3ze3; Li, Lyons *et al.*, 2013 ▶). (*e*) Crystals of SeMet-labelled CM41 are shown in (i). The anamalous map (magenta) calculated using diffraction data collected at the selenium edge (0.97944 Å) at 4 r.m.s.d. is viewed parallel to the membrane (ii) and from the periplasm (iii). Only one trimer in the asymmetric unit is shown.

In the process of omitting individual components from the five-component reference condition above, a new rectangular crystal form of CM41 was obtained (Fig. 11 ▶ *a*). Surprisingly, nitrate, which was critical for the growth of bipyramid crystals and had been considered to be key to the success to date of the now three-year project, inhibited the formation of the new crystal form (Figs. 8 ▶ *a* and 11 ▶ *a*). The latter grew to full size in about four weeks and diffracted reproducibly to high resolution, with the best data set complete to 2.05 Å resolution.

The results just presented highlight the importance of (i) performing crystallization screens that cover a broad range of conditions and (ii) being open-minded and willing to re-evaluate conditions long after they have been accepted as integral to the success of the project. While crystallization can be rationalized to some degree, it still remains largely empirical. Rationalization may provide a direction and an overall strategy for a crystallization project. However, the exact protocol that leads to high-quality crystals still must be worked out experimentally by trial and error.

### 3.10. *De novo* phasing for DgkA structure determination   

Since the new rectangular crystals of CM41 diffracted to high resolution, the focus immediately shifted to this crystal form. Three approaches were taken, essentially in parallel, to obtain experimental phases. Crystals from the same batch as the highly diffracting crystals were soaked with Ta_6_Br_12_ and turned green (Fig. 11 ▶ *b*). The crystals were much darker in colour than the hosting mesophase, suggesting that the Ta_6_Br_12_ cluster had bound to the protein. The labelled crystals provided a 3.2 Å resolution data set. An anomalous signal to 6 Å resolution with a fourfold anomalous redundancy was observed. These data provided some low-resolution phasing, but not enough to satisfactorily solve the structure.

The second approach involved pre-labelling CM41 with EMP and CH_3_HgCl. Crystals with the same morphology as, and of a comparable size to, those of the unlabelled protein were obtained (Figs. 11 ▶ *c* and 11 ▶ *d*) that diffracted to 2.5 Å resolution. Crystallographic analyses did not reveal a usable anomalous signal and so these crystals were not followed up.

The third approach employed SeMet labelling of CM41 and a Met-auxotrophic *E. coli* strain B834 (DE3) to express the protein. MS analysis showed a peak corresponding to a molecular weight of 14 281.20 Da consistent with two selenium sites per protein monomer, presumably at Met63 and Met66 (Table 2 ▶). No detectable peaks were observed for the unlabelled protein, indicating a high labelling efficiency. The SeMet protein was as enzymatically active as the native CM41 and crystallized in the same condition as the native protein (Fig. 11 ▶ *e*). A highly redundant data set (100-fold anomalous redundancy) to 2.95 Å resolution, obtained by merging data collected from 18 SeMet CM41 crystals (Li, Lyons *et al.*, 2013 ▶), yielded a beautifully phased map which allowed automatic tracing of the majority of the sequence. Phases and a readily interpretable map could also be achieved initially using one third of the SeMet data. However, the more redundant data set gave a better quality map.

## 4. Concluding remarks   

**Figure 12:**
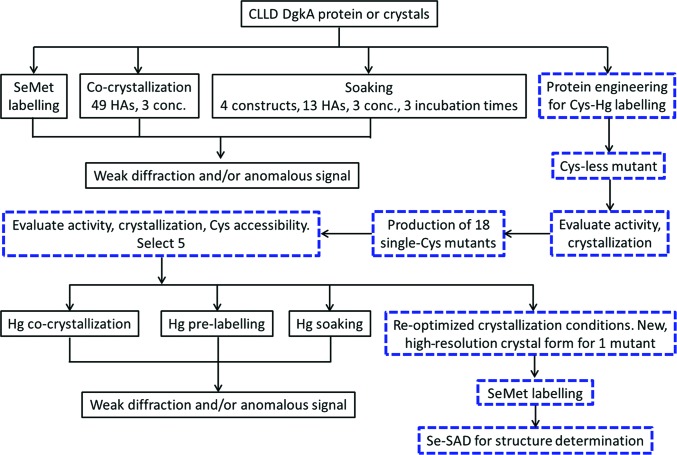
A flow chart of the steps taken to experimentally phase DgkA using crystals grown by the *in meso* method. The blue dashed boxes identify the route taken to solve the phase problem. Exact details are included in the text and in Table 1 ▶. Abbreviations used: conc., concentrations; HA, heavy atom; Hg, mercury.

Because a solution NMR structure was available at the time (Van Horn *et al.*, 2009 ▶), solving the structure of DgkA was expected to be a relatively straightforward task involving molecular replacement once initial crystals that diffracted to 3.7 Å resolution had been obtained. However, after extensive molecular-replacement trials (not discussed here) failed, we embarked on a challenging journey to solve the phase problem experimentally. SeMet crystals of the first crystal form were unfortunately too weakly diffracting to be useful. Our next approach involved heavy-atom co-crystallization, which was chosen because it can be implemented easily and does not require crystal manipulation. However, this was mostly unsuccessful in generating crystals. A protocol for heavy-atom soaking of crystals in the cubic phase was developed next and explored thoroughly. Working with small, radiation-sensitive crystals that exhibited background mesophase scattering and diffraction of varying strengths meant it was often difficult to collect a complete data set. Anomalous signal, when found, was always weak either because the heavy atom was not ordered in the crystal or because the multiplicity in the data set required to measure it accurately could not be obtained. Further, data were often non-isomorphous and were not readily merged and scaled together. We then embarked on generating a series of site-specific single-cysteine mutants for mercury labelling in the hope the heavy atom could be placed confidently in a well ordered part of the protein. However, the crystals obtained diffracted poorly. One mutant was observed to produce a crystal form not previously observed in the study thus far that resulted in a 2.05 Å resolution native data set. SeMet labelling led to reasonably well diffracting crystals and to a relatively straightforward SAD structure solution (Li, Lyons *et al.*, 2013 ▶). The entire process is summarized in Fig. 12 ▶.

With the structure in hand, we have reviewed some of the data collected *en route* to a final structure solution in order to understand what did or did not happen in the course of our failed attempts at heavy-atom labelling by soaking, pre-labelling and co-crystallization. For the CLLD and CM41 constructs the solved structures (PDB entries 3ze5 and 3ze3) were used to phase the best data sets (based on completeness and resolution) collected in the presence of heavy atoms, either by co-crystallization or soaking, for each heavy atom. Anomalous and difference maps were then calculated using *PHENIX* (Adams *et al.*, 2010 ▶) and inspected in *Coot* (Emsley & Cowtan, 2004 ▶). Where anomalous/difference density was observed, the corresponding heavy atom was positioned and its occupancy was refined. The results of this analysis are summarized in Table 3 ▶. Briefly, K_2_PtCl_4_ soaked into CLLD crystals showed four sites per asymmetric unit of very low occupancy (10–16%). Ta_6_Br_12_ (tantalum bromide) soaked into CLLD crystals showed two sites in which single Ta atoms were positioned and refined to occupancies of 23 and 53%. The density did not support a full cluster and so it must be assumed that the occupancy of the cluster was very low. None of the other heavy atoms were present in the CLLD crystals. The Ta_6_Br_12_ soak of rectangular CM41 crystals revealed density for a cluster on the threefold axis at the periplasmic side of each trimer in the asymmetric unit (Fig. 11 ▶ *b*). Individual clusters are coordinated by three aspartates: one (Asp51) from each monomer. These clusters refine with occupancies of 40 and 50% (Table 3 ▶). Pre-labelling with mercury in CM41 revealed anomalous density at Cys41 in chains *A*, *B* and *D*, as well as where a zinc ion was modelled in the CM41 structure (PDB entry 3ze3), with refined occupancies of 19–38% (Table 3 ▶). The anomalous diffraction data from the CM41–EMP crystals were collected at 12.32 keV, which is at the high-energy remote of the Zn peak (9.68 keV). Zn still scatters at 12.32 keV. Therefore, the possibility of this site being a Zn cannot be ruled out. There was no significant anomalous density at any of the methionine residues. We find the low occupancy surprising given the battery of experiments prior to crystallization that suggested accessibility and therefore a high likelihood of labelling. In this crystal form, however, some of the Cys41 residues are buried at protein–protein contact sites in the crystal and some are in less ordered regions of the structure. Specifically, the first transmembrane helix, in which Cys41 is located, in chains *E* and *F* is less well defined in density than the rest of the structure. Still, if highly redundant data were collected for the CM41–EMP crystals it might be possible to obtain sufficient phases for solving the structure.

To conclude, the key to solving the structure of DgkA was to obtain a different crystal form and to use it in conjunction with SeMet labelling. The new form came about serendipitously in the process of generating a series of single-cysteine mutants originally designed for mercury labelling. Upon reflection, it appears to us that a screen of mutants, by means of an alanine scan, for example (Shibata *et al.*, 2013 ▶), could provide a way to generate different crystal forms for a given membrane-protein target in the lipid mesophase. These could then be used to optimize for diffraction quality and subsequently be used for SeMet labelling. However, for protein targets that do not lend themselves conveniently to SeMet labelling or to mutant screening (proteins from a native source, for example) traditional heavy-atom soaking should be tried in the knowledge that doing so *in meso*, as described here, is technically challenging, time-consuming and not terribly efficient. Despite its size, the Ta_6_Br_12_ cluster diffuses readily throughout the cubic mesophase and may be a good heavy-atom label with which to embark on a campaign of derivatization. And it is green!

## Figures and Tables

**Table 1:** Summary of heavy-atom labelling of DgkA for crystal structure determination

	Soaking†		Pre-labelling‡		
Co-crystallization§	9.9 MAG	7.8 MAG	HgCl_2_, HgAc	EMP, CH_3_HgCl	SeMet
Hg (11): mersalyl acid, HgCl_2_, HgAc, thiomersal, K_2_(HgI_4_), HgBr_2_, Hg(NO_3_)_2_, Hg(CN)_2_, CH_3_HgCl, EMP¶, PCMB¶	Mersalyl acid (0.10.5m*M*) [4h2d] (CLLD, CM46††, CM53††)	HgCl_2_ (110m*M*) [16h] (CLLD, CM46)	CM46	CM41††	CLLD
	HgCl_2_ (0.015m*M*) [12h] (CM41, CM46)	EMP (1m*M*) [412h] (CM46, CM53)		CM42††	CM41
Pt (11): K_2_PtCl_4_, (NH_4_)_2_PtCl_4_, K_2_PtCl_6_, K_2_Pt(NO_2_)_4_, K_2_Pt(CN)_4_, PtCl_2_(H_2_NCH_2_CH_2_CH_2_NH_2_), Pt(NH_3_)_2_(NO_2_)_2_, K_2_PtBr_4_, K_2_PtBr_6_, K_2_PtI_6_, K_2_Pt(CNS)_6_	HgAc (1m*M*) [4h] (CM46)	K_2_PtCl_4_ (10m*M*) [5h] (CM46)		CM43††	
	EMP (15m*M*) [412h] (CM46)	Sm(NO_3_)_3_ (10m*M*) [5h] (CM46)		CM46	
	PCMB (1m*M*) [12h] (CM46)	Ta_6_Br_12_ (35700*M*) [216h] (CM41, CM46)		CM53	
	K_2_PtCl_4_ (2.525m*M*) [120h] (CLLD)				
Au (5): KAu(CN)_2_, NaAuCl_4_, AuCl_3_, HAuCl_4_, KAuBr_4_	Sm(NO_3_)_3_ (10m*M*) [20h] (CLLD)				
	GdCl_3_ (2.5m*M*) [2h] (CLLD)				
Others (21): Na_2_WO_4_, Sm(NO_3_)_3_, La(NO_3_)_3_, Eu(NO_3_)_3_, GdCl_3_, LuCl_3_, YbCl_3_, DyCl_3_, PrCl_3_, NdCl_3_, HoCl_3_, K_2_ReCl_6_, TlCl_3_, TlCl, Pb(NO_3_)_2_, AgNO_3_, CdCl_2_, K_2_IrCl_6_, K_2_OsO_4_, CH_3_CO_2_Pb(CH_3_)_3_ (Pb, TMLA¶), Ta_6_Br_12_	Pb(NO_3_)_2_ (210 m*M*) [120h] (CLLD)				
	TMLA (100m*M*) [6h] (CLLD)				
	NaAuCl_4_ (2.510m*M*) [2h] (CLLD)				
	K_2_AuCl_4_ (10m*M*) [56h] (CM46)				
	Ta_6_Br_12_ (10140*M*) [616h] (CLLD)				

† Under Soaking, table entries are arranged as follows: heavy-atom identity (concentration range) [soaking time range] (construct). Typically three concentrations and four different times were used in the indicated ranges.

‡ Crystallization of pre-labelled mutants were mostly carried out using 7.8 MAG as the host lipid.

§ Co-crystallization was carried out using both 7.8 MAG and 9.9 MAG as host lipids. The concentrations screened were 40, 100, 200 and 500*M*. For Sm(NO_3_)_3_ and Pb(NO_3_)_2_, additional concentrations of 1, 1.2, 1.5 and 2m*M* were tested.

¶ Abbreviations: EMP, ethylmercury phosphate; PCMB, *p*-chloromercuribenzoic acid; TMLA, trimethyllead acetate; HgAc, mercury acetate.

†† Construct nomenclature is described in [Sec sec3.5]3.5. Single-Cys mutants are based on the Cys-less mutant (C46A, I53V, I70L, M96L, V107D and C113A). The number in each single-Cys mutant identifier identifies the sequence position of the engineered cysteine residue.

**Table 2:** MS analysis of DgkA

Sample	Attachment	Expected MW† (Da)	Observed MW‡ (Da)	Expected MW difference§ (Da)	Observed MW difference¶ (Da)
CM41	None	14187.48	14186.80 (80)	0	0.68
	DM (1)	14670.05	14669.58 (20)	482.57	482.10
CM41DTNB	NTB^2^	14384.66	14384.45	197.18	196.97
CM41EMP	None	14187.48	14186.89 (39)	0	0.59
	EMP (1)	14416.13	14415.83 (31)	228.65	228.35
	EMP (2)	14644.79	14644.45 (25)	457.31	457.76
	EMP (1), DM (1)	14898.70	14898.84 (5)	711.22	711.36
CM41CH_3_HgCl	None	14187.48	14187.02 (43)	0	0.46
	CH_3_HgCl (1)	14402.11	14402.07 (39)	214.63	214.59
	CH_3_HgCl (2)	14616.73	14616.93 (15)	429.25	429.91
	DM (1), CH_3_HgCl (1)	14884.68	14884.41 (5)	697.20	696.93
CM41-SeMet	SeMet	14281.27	14281.61 (70)	93.79	94.13
	SeMet, DM (1)	14763.84	14764.19 (30)	576.36	576.71
CM43	None	14159.42	14159.13 (76)	0	0.29
	DM (1)	14641.99	14642.16 (17)	482.57	482.74
	DM (2)	15124.56	15124.43 (5)	965.14	965.01
	DM (3)	15607.13	15607.91 (2)	1447.71	1448.49
CM43DTNB	NTB^2^ (1)	14356.60	14356.98 (90)	197.18	197.56
	NTB^2^ (1), DM (1)	14839.17	14839.20 (10)	679.75	679.78
CM43EMP	None	14159.42	14158.77 (35)	0	0.65
	EMP (1)	14388.07	14388.15 (23)	228.65	228.73
	EMP (2)	14616.73	14616.54 (20)	457.31	457.12
	DM (1)	14641.99	14642.72 (7)	482.57	483.30
	DM (1), EMP (1)	14870.64	14870.78 (5)	711.22	711.36
	DM (1), EMP (2)	15099.29	15099.19 (4)	939.87	939.77
	DM (1), EMP (1)	15353.21	15352.85 (2)	1193.79	1193.43
	DM (2), EMP (2)	15581.86	15580.00 (1)	1422.44	1420.58
	DM (3)	15607.13	16606.40 (1)	1447.23	1446.98
CM46	None	14187.48	14187.19 (70)	0	0.29
	DM (1)	14670.05	14669.70 (20)	482.57	482.22
	DM (2)	15152.62	15152.00 (8)	965.14	964.52
	DM (3)	15635.19	15635.00 (2)	1447.71	1447.52
CM46DTNB	NTB^2^ (1)	14384.66	14384.88 (80)	197.18	197.40
	NTB^2^ (1), DM (1)	14867.23	14867.06 (16)	679.75	679.58
	NTB^2^ (1), DM (2)	15349.80	15349.30 (4)	1162.32	1161.82
CM46EMP	None	14187.48	14187.71 (49)	0	0.23
	EMP (1)	14670.05	14416.65 (20)	228.65	229.17
	EMP (2)	14644.79	14645.15 (17)	457.31	457.67
	DM (1)	14670.05	14671.15 (9)	482.57	483.67
	DM (1), EMP (1)	14898.70	14899.78 (2)	711.22	712.30
	DM (1), EMP (2)	15127.35	15127.60 (2)	939.87	940.12
	DM (2)	15152.62	15153.59 (1)	965.14	966.11

† The expected MW of nonlabelled protein was calculated based on the amino-acid composition of the DgkA construct with the N-terminal Met missing as a result of processing *in vivo* (Frottin *et al.*, 2006 ▶; Hopper *et al.*, 2013 ▶). The expected MWs for mercury/Ellman’s reagent modifications were calculated based on a reaction in which the added chemical groups replace the hydrogen of the thiol group. For SeMet-labelled protein, the value was calculated by replacing the S atom in the two methionine residues with selenium.

‡ The values in parentheses are the percentages of each species, which were calculated based on peak height, assuming that the samples with and without labelling behave in the same manner during the MS experiment.

§ Refers to the MW difference between the labelled and unlabelled protein.

¶ The MW difference was calculated using the observed MW subtracted from the theoretical MW of the unlabelled protein.

**Table 3:** Retrospective analysis of heavy-atom derivatization data based on co-crystallization, soaking and pre-labelling trials

Construct/lipid	Heavy atom	Treatment	Resolution ()	Occupancy for heavy-atom sites†
CLLD/9.9 MAG	Pb(NO_3_)_2_	Co-crystallization, 125*M*	3.7	N/A†
	Sm(NO_3_)_3_	Co-crystallization, 60*M*	3.6	N/A
	Sm(NO_3_)_3_	Co-crystallization, 0.5m*M*	7.1	N/A
	Mersalyl acid	Co-crystallization, 12*M*	3.3	N/A
	Pb(NO_3_)_2_	Soak, 2m*M*, 20h	3.75	N/A
	Sm(NO_3_)_2_	Soak, 10m*M*, 20h	3.55	N/A
	Mersalyl acid	Soak, 0.5m*M*, 4h	3.46	N/A
	HgCl_2_	Soak, 2.5m*M*, 2h	6.7	N/A
	K_2_PtCl_4_	Soak, 5m*M*, 4h	3.6	0.1, 0.13, 0.14, 0.16
	NaAuCl_4_	Soak, 2.5m*M*, 1h	3.45	N/A
	TMLA	Soak, 2.5m*M*, 2h	3.4	N/A
	GdCl_3_	Soak, 2.5m*M*, 2h	3.0	N/A
	Ta_6_Br_12_	Soak, 0.1m*M*, 4h	3.45	0.53, 0.23‡
CM41/7.8 MAG	Ta_6_Br_12_	Soak, 0.7m*M*, 7h	3.2	0.4, 0.5§
	EMP	Pre-labelled	2.8	0.19, 0.26, 0.27, 0.38

† Anomalous and difference maps were phased using the relevant structures of DgkA [PDB entries 3ze5 (CLLD) and 3ze3 (CM41)]. Heavy atoms were placed in the structures with reference to the anomalous maps. Occupancies were refined using *phenix.refine*. Values shown refer to individual sites in an asymmetric unit.

‡ No anomalous peaks were identified.

§ Single Ta atom only; the density would not support a cluster.

¶ Ta_6_Br_12_ cluster refined.
